# Norbixin Protects Retinal Pigmented Epithelium Cells and Photoreceptors against A2E-Mediated Phototoxicity *In Vitro* and *In Vivo*

**DOI:** 10.1371/journal.pone.0167793

**Published:** 2016-12-16

**Authors:** Valérie Fontaine, Elodie Monteiro, Elena Brazhnikova, Laëtitia Lesage, Christine Balducci, Louis Guibout, Laurence Feraille, Pierre-Paul Elena, José-Alain Sahel, Stanislas Veillet, René Lafont

**Affiliations:** 1 Sorbonne Universités, UPMC Univ Paris, INSERM, CNRS, Institut de la Vision, 17 Rue Moreau, Paris, France; 2 Biophytis, Parc BIOCITECH, 102 Avenue Gaston Roussel, Romainville, France; 3 IRIS-Pharma, Les Nertières, Allée Hector Pintus, La Gaude, France; Indiana University School of Medicine, UNITED STATES

## Abstract

The accumulation of *N*-retinylidene-*N*-retinylethanolamine (A2E, a toxic by-product of the visual pigment cycle) in the retinal pigment epithelium (RPE) is a major cause of visual impairment in the elderly. Photooxidation of A2E results in retinal pigment epithelium degeneration followed by that of associated photoreceptors. Present treatments rely on nutrient supplementation with antioxidants. 9’-*cis*-Norbixin (a natural diapocarotenoid, 97% purity) was prepared from *Bixa orellan*a seeds. It was first evaluated in primary cultures of porcine retinal pigment epithelium cells challenged with A2E and illuminated with blue light, and it provided an improved photo-protection as compared with lutein or zeaxanthin. In *Abca4*^*-/-*^ *Rdh8*^*-/-*^ mice (a model of dry AMD), intravitreally-injected norbixin maintained the electroretinogram and protected photoreceptors against light damage. In a standard rat blue-light model of photodamage, norbixin was at least equally as active as phenyl-N*-tert*-butylnitrone, a free radical spin-trap. Chronic experiments performed with *Abca4*^*-/-*^ *Rdh8*^*-/-*^ mice treated orally for 3 months with norbixin showed a reduced A2E accumulation in the retina. Norbixin appears promising for developing an oral treatment of macular degeneration. A drug candidate (BIO201) with 9’-*cis*-norbixin as the active principle ingredient is under development, and its potential will be assessed in a forthcoming clinical trial.

## Introduction

In developed countries, AMD is the major cause of blindness in the elderly [1]. Dry AMD, the most frequent form, is a slowly evolving pathology. Early dry AMD is characterized by retinal deformation owing to the local accumulation of waste deposits, then photoreceptors degenerate in small areas, which increase in size, leading to advanced dry AMD (geographic atrophy).

Photoreceptor death in the central part of the retina (the macula) is responsible for the loss of high-resolution colour vision. It follows the death of the retinal pigment epithelium (RPE) cells [2], which have major roles in the visual pigment cycle and the phagocytosis of shed oxidized photoreceptor outer segments. The A2E-induced disturbance of RPE cell lysosomal activity supposedly represents one early stage of AMD development and correlates with the accumulation of A2E and related retinal dimers [3,4].

A2E and its isomers are formed by the reaction of two *trans*-retinal molecules with phosphatidyl-ethanolamine [3]. A2E is an amphiphilic molecule, which can alter membrane properties [5] and impair lysosomal functions [6,7]; A2E can activate inflammatory processes by recruiting macrophages [8], increase VEGF secretion *in vitro* [8,9] and possibly also promote neovascularization through enhanced vascular endothelial growth factor production [10].

High A2E concentrations increase oxidative stress in RPE cells *in vitro* [7]. In the presence of blue light and oxygen, A2E undergoes photooxidation as evidenced by the appearance of toxic oxygen adducts [11,12]. It generates small amounts of singlet oxygen [13,14,15] and is finally cleaved to small reactive aldehydes [16,17,18,19], which contribute to its deleterious effects. Accumulation of damaged protein in RPE cells is directly associated with AMD development [20]. A2E photo-oxidation products also damage DNA [21] and activate the complement system [22]. The toxic activity of A2E photodegradation products was evidenced by incubating RPE primary cell cultures in the dark with a previously illuminated solution of A2E [23].

Given the direct involvement of A2E in the pathology, several strategies have been considered for designing treatments, either by preventing the formation/accumulation of this molecule, or by counteracting/reducing its deleterious effects [24,25,26,27,28]. Such attempts include (list not exhaustive):

1. reducing A2E formation by retinylamine, a visual pigment cycle inhibitor [29], by reducing retinol supply to the retina by a RBP4 inhibitor [30] or by feeding deuterium-enriched vitamin A, which shows a reduced conversion into A2E [31];
2. promoting A2E removal by intravitreal injection of cyclodextrins [32,33] or by promoting enzymatic degradation of A2E [34,35];
3. reducing A2E oxidation by feeding natural antioxidants (carotenoids, flavonoids, resveratrol, etc.) [36,37,38] or synthetic ones, e.g. PBN derivatives [39];
4. counteracting some A2E direct effects, such as treatments for a re-acidification of lysosomes [40,41], or treatments aimed to restore efficient autophagic processes [42];
5. counteracting long-term consequences of A2E accumulation (drusen formation) by using inhibitors/antibodies to complement alternative pathway [43].

Despite promising results, none of these approaches has resulted up to now in a recognized efficient treatment of dry AMD. Current treatments have a limited efficacy and and rely on nutritional formulae containing zinc, various antioxidants (vitamins C and E) and carotenoids, whose components have been tested either individually or as various mixtures over several years (AREDS 1 and 2 studies). These studies have indeed established the protective role of zinc and, for a part of the population tested, of carotenoids [44,45].

The rationale for using specific carotenoids (lutein, zeaxanthin and meso-zeaxanthin) relies on the fact that they are naturally present in the macula. As with other antioxidants, their biological activity is not limited to an antioxidant effect [46,47]. They are expected to act there as filters for blue light [48], as antioxidants [49], to display anti-inflammatory properties [50] and to attenuate A2E formation [36].

**Fig 1:**
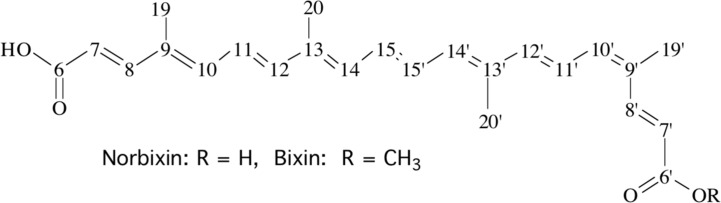
Structural formulae of bixin and norbixin. These two 6,6’-diapocarotenoids are represented here as the 9’-*cis* isomers used in the present study. They may theoretically occur as many isomers, as each double bond may be either *cis* or *trans*.

We previously observed the photo-protective activity of two diapocarotenoids, bixin and norbixin (Fig 1), for skin cells against UVB [51] and these data prompted us to engage in a programme for assessing the potential of these molecules for the treatment of dry AMD. For that purpose, we used specific *in vitro* tests with RPE primary cultures from porcine retina and a set of *in vivo* experiments to assess the efficiency of these molecules, as described below.

## Materials and Methods

### Animals

For blue-light-induced retinal damage (BLD) studies pigmented *Abca4*^*-/-*^ *Rdh8*^*-/-*^ [52] (used under licence from Case Western Reserve University to Biophytis) and *Abca4*^*+/+*^ *Rdh8*^*+/+*^ mice both carrying the Rpe65-Leu450 mutation and the *rd8* mutation in the *Crb1* gene were used. *Abca4*^*+/+*^ *Rdh8*^*+/+*^ mice were derived from backcrossing *Abca4*^*-/-*^ *Rdh8*^*-/-*^ and C57Bl/6N mice (Janvier Labs, Le Genest-Saint-Isle, France). Pigmented C57BL/6J mice (25–30 g) were provided by Harlan Laboratories (Gannat, France) and used in pharmacokinetics studies. Male Sprague-Dawley rats (240–320 g) were provided by Charles River (Saint Germain Nuelles, France) and used for BLD. Animals were housed under 12-hour on/off cyclic normal lighting.

### Ethics statement

All procedures were carried out according to the guidelines on the ethical use of animals from the European Community Council Directive (86/609/EEC) and were approved by the French Ministry of Agriculture (OGM agreement 6193) and by the Committee on the Ethics of Animal Experiments of the French Ministry of Research. All efforts were made to minimize suffering.

### Reagents/Chemicals

All usual chemicals were from Sigma (St. Louis, MO, USA). Reagents for cell culture were from Thermo Fisher Scientific (Waltham, MA, USA). Ketamine, xylasine, tropicamide and oxybuprocaine chlorhydrate were from Centravet (Maison-Alfort, France). Optimal cutting temperature compound and other reagents for histology were from Roth Sochiel (Lauterbourg, France).

Products for the synthesis of A2E or analogues (retinaldehyde, ethanolamine, propylamine) were supplied by Sigma. Reference carotenoids—lutein, zeaxanthin, crocetin—were from Extrasynthèse (Genay, France). 9’-*cis*-Bixin (AICABIX P, purity 92%) extracted from *Bixa orellana* seeds was purchased from Aica-Color (Cusco, Peru). 9’-*cis*-Norbixin was prepared from bixin upon alkaline hydrolysis according to Santos et al. [53]. Bixin (10 g) was weighed in a 100 mL capped bottle and solubilized with 28 mL ethanol and homogenized using an ultrasonic bath; 72 mL of a 5% (w/v) KOH solution in water was added to the previous mixture before being placed on a hot-plate stirrer at 55°C for 5 days in the dark. The progress of the reaction was followed by high-performance liquid chromatography (HPLC) analyses. Once bixin was totally converted to norbixin, the reaction mixture was transferred to a Florentine flask and ethanol was evaporated using a rotary evaporator. The KOH still present in the reaction mixture was neutralized using 8 mL acetic acid, which induced norbixin precipitation. The solid paste obtained was placed on a fritted glass filter funnel and rinsed with 2 L 0.1% (v/v) trifluoroacetic acid solution to ensure the lack of any salt form of norbixin. This acidified paste was placed in another Florentine flask and suspended in 40 mL 0.1% trifluoroacetic acid solution in water. The suspension was then frozen using liquid nitrogen before being freeze-dried over 2 days. The obtained product (the 9’-*cis* isomer) showed a HPLC purity of 97% as confirmed by ^1^H-nuclear magnetic resonance (using malonic acid as internal standard).

### *In vitro* model of RPE phototoxicity and treatments

Pig eyes were obtained from a local slaughterhouse and transported to the laboratory in ice-cold Ringer solution. After removal of the anterior segment of the eye, the vitreous and neural retina were separated from the RPE and removed. The eyecup was washed twice with phosphate buffer saline (PBS), filled with trypsin (0.25% in PBS) and incubated at 37°C for 1.5h. RPE cells were harvested by gently pipetting, centrifuged to remove trypsin and re-suspended in Dulbecco’s Modified Eagle Medium (DMEM) supplemented with 20% (v/v) foetal-calf serum (DMEM20%FCS) and 0.1% gentamycin. Cells were seeded into 60 mm diameter Petri dishes, cultured in an atmosphere of 5% CO_2_/95% air at 37°C, and supplied with fresh medium after 24 hours and 4 days *in vitro*. After one week in culture, cells were trypsinized and transferred to a 96-well plate at a density of 1.5 x 10^5^ cells/cm^2^ in DMEM2%FCS. After 2 days *in vitro*, A2E was added to the medium at a final concentration of 30 μM, and 19 hours later blue-light illumination was performed for 50 minutes using a 96 blue-led device (Durand, St Clair de la Tour, France) emitting at 470 nm (1440 mcd, 8.6 mA). Just before illumination, the culture medium was replaced by a modified DMEM without any photosensitizer and with 2% FCS. 24 hours after blue-light irradiation, all cell nuclei were stained with Hoechst 33342 and nuclei of dead cells with ethidium homodimer 2, fixed with paraformaldehyde (4% in PBS, 10 min) and 9 pictures per well were captured using a fluorescence microscope (Nikon TiE) equipped with a CoolSNAP HQ2 camera and driven by Metamorph Premier On-Line program. Quantification of live cells was performed using Metamorph Premier Off-Line and a home-made program by subtraction of dead cells from all cells.

Cell treatments were performed as followed. All the drugs used in these experiments were prepared as stock solutions in DMSO. Drugs tested for their protective effect were added to the culture medium 48 hours before illumination.

**Fig 2:**
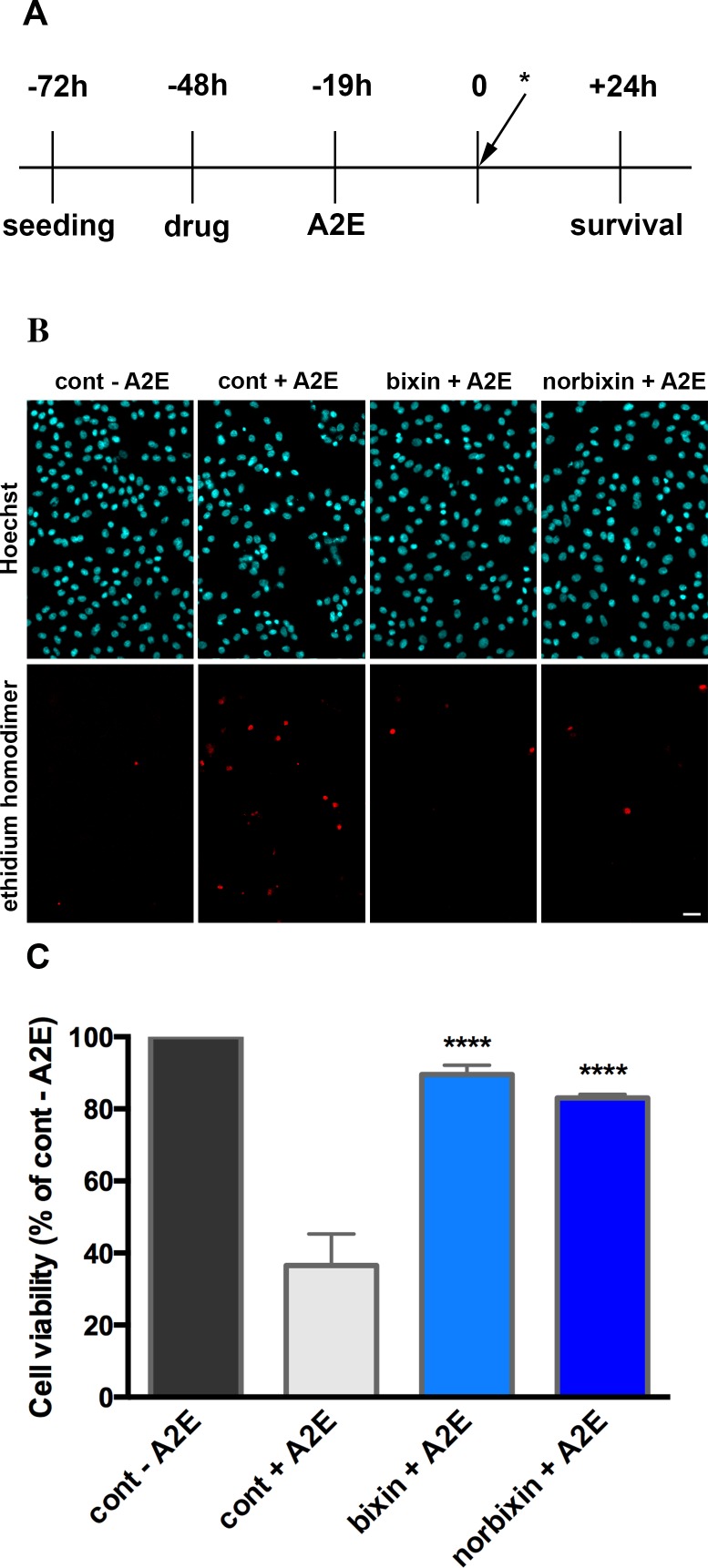
Bixin and norbixin protect RPE cells from A2E-induced blue-light phototoxicity. (A) Steps of RPE cell treatment in the *in vitro* model of phototoxicity. * indicates blue-light illumination (B) Representative pictures of RPE cell nuclei stained with Hoechst 33342 and ethidium homodimer 2 24 hours after illumination. In the controls (cont), cells were treated with DMSO instead of bixin and norbixin, then either treated or not with A2E. Bixin and norbixin treatments were performed 48 hours before illumination. A2E treatment was done 19 hours before illumination. Pictures were taken in the centre of a well (scale bar = 20 μm). (C) Bixin and norbixin effects on RPE cell survival after A2E + illumination compared to non-treated cells (cont—A2E) or cells treated with A2E only (cont + A2E). Data in B and C are representative of five independent experiments with *n* = 4. Bars represent mean +/- s.e.m. ****p<0.0001 compared to cont + A2E (One-way ANOVA, Dunnett’s post test).

For experiments aimed at measuring A2E in RPE in culture cells were seeded in 12-well plates and the treatments were performed as before (see Fig 2A), but the experiment was stopped before illumination. Cells were collected in Eppendorf tubes, frozen in liquid nitrogen and kept at -80°C until analysis.

### *In vivo* drug administration

#### Intravitreal treatment

Two groups of 14 *Abca4*^*-/-*^ *Rdh8*^*-/-*^ mice aged 7 weeks were used for intravitreal injections. Twenty-four hours before BLD, one group received an intravitreal injection of 2 μL of 500 μM norbixin (in 0.3% DMSO) in one eye. The vitreous volume of the mouse eye being 5.3 μL [54] we estimated that the final concentration of norbixin in the vitreous was approximately 130 μM. The other group received an intravitreal injection of 2 μL of 0.3% DMSO in PBS in one eye. Injections were performed using sterile 33-gauge blunt tip needles connected to an UMP-2D injector and a Micro4™ controller (World Precision Instruments, Sarasota, FL, USA).

#### Intraperitoneal treatment

Rats were randomly assigned to non-induced control or light-exposed groups (n = 6 per group). Rats were injected intraperitoneally with either norbixin (10, 50, 100 mg/kg), phenyl-N*-tert*-butylnitrone (PBN, 50 mg/kg), a potent free-radical trapping agent, or an equivalent volume of vehicle (water/tetraglycol/DMSO, 70:20:10 v/v/v) 30 min prior to light damage and 2, 4 and 6 hours after the beginning of the exposure.

#### Norbixin-supplemented beverage

In order to test the preventive action of norbixin, two groups of 10 *Abca4*^*-/-*^ *Rdh8*^*-/-*^ mice, aged 2 months, received norbixin orally in drinking water for 3 months. Since the product is poorly soluble in water when the pH is neutral, an exact amount of 50 mg was weighed into 15 mL Falcon tubes and first dissolved in 2 mL DMSO; then 2 mL Tween 80 were added and the mixture was transferred in feeding bottles and adjusted up to 100 mL with water. The mice absorbed *ca*. 5 mL water daily, corresponding to a dose of 2.5 mg norbixin/day. *Abca4*^*-/-*^ *Rdh8*^*-/-*^ mice aged two and five months were used as non-treated controls. After 3 months of supplementation, mice were euthanized and eyes were removed for A2E measurements.

### *In vivo* BLD

A custom-made light damage device equipped with fluorescent lamps (Phillips TL-D 36W/18) with UV filter was used to induce BLD in mice (Durand, St-Clair de la Tour, France). All manipulations with the animals were performed in dim red light. Pupils were dilated with 0.5% tropicamide eye solution before illumination. Mice, previously maintained in a 12-hour light (≈ 10 lux)/ 12-hour dark cycle environment for two weeks, were dark-adapted for 24 hours. Rats were dark-adapted for 36 hours. Light damage was induced at 4000 lux for 1 hour for mice and 6 hours for rats. Following exposure to light damage, animals were placed in the dark for 24 h and then returned to the dim cyclic light environment for 7 days.

#### Full-field electroretinogram (ERG)

ERG recordings were performed with the Electroretinograph Toennies Multiliner Vision designed for rodents. ERG was performed one week after BLD in mice and rats. After overnight dark adaptation, mice and rats were anesthetized with ketamine (100 mg/kg) and xylazine (10 mg/kg). Eye drops were used to dilate the pupils (0.5% tropicamide) and anesthetize the cornea (0.4% oxybuprocaine chlorhydrate). Body temperature was maintained at 37°C using a circulating hot-water heating pad. Corneal electrodes (Ocuscience, a subsidiary of Xenolec Inc., USA) were placed on the corneal surface of each eye. Eye gel (Lubrithal, Dechra Pharmaceuticals, Northwich, UK) was used to maintain good contact and corneal moisture. Needle electrodes placed subcutaneously in cheeks served as reference and a needle electrode placed in the back served as earth. The ERG was recorded from both eyes simultaneously after placing the animal into the Ganzfeld bowl. Five responses to light stimulus at 10 cd.s.m^-2^ were averaged. Amplitudes of a- and b-waves of mixed rod and cone response were determined.

### Histology and photoreceptor counting

After ERG, animals were euthanized and eyes were enucleated and dissected to remove the cornea and lens. For mice, eyes were fixed in 4% paraformaldehyde/5% sucrose (in PBS) for one hour at 4°C. The eye cups were then cryoprotected by successive bathing in 5% sucrose (1h), 10% sucrose (1h) and 20% sucrose (overnight), embedded in optimal cutting temperature compound, and cryosections (10 μm) were prepared using Superfrost® Plus slides and stored at -20°C until analysis. Sections were stained with Hoechst 33342 to label nuclei and were scanned using a nanozoomer (NDP.scan v2.5.86, Hamamatsu, Japan) with fluorescence imaging modules. Photoreceptor nuclei were quantified at 200 μm intervals superior and inferior to the edge of the optic nerve head along the vertical meridian using the NDP.view software.

Rat eyes were fixed with Hollande’s Bouin fixative solution for at least 48 hours at room temperature. The cornea and lens were removed from the eyeballs. The fixed tissues were then embedded in paraffin. Sections (5 to 7 μm thick) were performed along the vertical meridian and stained with Trichrome-Masson. Digitized images of fourteen locations (seven in the superior retina 250–3250 μm above the optic disc and seven in the inferior retina 250–3250 μm below the optic disc) were obtained for each section using a Digital Imaging system (LAS system; Leica). The number of photoreceptor cell nuclei in each image was counted on each photograph.

### Synthesis of A2E and A2E-propylamine

A2E (*N*-retinylidene-*N*-retinylethanolamine) was synthesized by Orga-link (Magny-Les-Hameaux, France) as described before [55]. Briefly, all-*trans*-retinal, ethanolamine and acetic acid were mixed in absolute ethanol in darkness at room temperature over 7 days. The crude product was purified by preparative HPLC in the dark to isolate A2E with a purity of 98% as determined by HPLC. A2E (20 mM in DMSO under argon) was stored at -20°C.

A2E-propylamine (an analogue of A2E) was synthesized using propylamine instead of ethanolamine and retinal according to the described procedure [56,57]. Briefly, 49.24 mg retinal were solubilized in a mixture containing 1.5 mL ethanol and 4.65 μΛ acetic acid. 1-Propylamine (6.33 μΛ) was then added to the mixture and the reaction occurred in 3 days at room temperature in the dark. The progress of the reaction was followed by analytical HPLC. At the end of the reaction, the compound was purified by preparative HPLC and thereafter used as internal standard for A2E quantification by HPLC coupled with tandem mass spectrometry (HPLC-MS/MS).

### A2E measurement by HPLC-MS/MS

Mass spectrometry provides accurate and sensitive quantification of A2E [58]. HPLC-MS/MS analysis was performed on an Agilent 1100 in-line triple quadrupole mass spectrometer (API365 or API3200, Applied Biosystems, Les Ulis, France) operated in MRM positive-ion mode. A2E was eluted on a reverse-phase C18 column (2.1x50 mm; 3.5 μm particle size; Symmetry, Waters, Guyancourt, France) with the following gradient of acetonitrile in water (containing 0.1% formic acid): 65–100% (4 min), 100% (5 min), (flow-rate: 0.3 mL/min). A2E-propylamine (25 ng) was used as internal standard. The AUC of A2E and A2E-propylamine were determined in MRM mode with precursor ion/product ion settings, A2E (592.5/105.1) and A2E-propylamine (590.6/186.2). For A2E quantification, a calibration curve was performed using various concentrations of A2E (5–10000 nM).

### A2E measurement in RPE cells or culture media

The concentration of A2E in RPE cells or culture media was determined with HPLC-MS/MS method described above. Samples were diluted with ACN or ACN/H_2_O (1:1, v/v) and the internal standard (A2E-propylamine) was added to each sample. The calibration curve of A2E was performed in ACN/H_2_O (1:1, v/v) and under these conditions, with 10 μL injections, the limit of quantification (LOQ) was 50 nM.

### A2E measurement in eyes

A2E present in eyes was determined with the HPLC-MS/MS method described above. Each eye was homogenized in CHCl_3_/MeOH (1:1, v/v) (0.5 mL) with homogenizer (Precellys-24) during 2 cycles (30 s) at 6500 rpm. The internal standard (A2E-propylamine) was added and the organic layer was extracted. The homogenate was then extracted two times with CHCl_3_/CH_2_Cl_2_ (0.5 mL). The combined organic extracts were dried *in vacuo* without heating (EZ2, Genevac Ltd Ipswich, U.K.). Then they were dissolved in 100 μL DMSO/MeOH (1:1, v/v) and transferred to microtitre plates. The calibration curve of A2E was prepared in CHCl_3_/MeOH (1:1, v/v) and dried *in vacuo* without heating (EZ2, Genevac), then dissolved in 100 μL DMSO/MeOH (1:1, v/v). Under these conditions, with an injection volume of 10 μL, the limit of quantification (LOQ) was 10 nM.

### Analysis of oxidized forms by MS (direct-inlet method)

Upon oxidation, A2E can fix between 1 and 9 oxygen atoms, each corresponding to an increase of molecular mass of 16 units [16]. The relative abundance of oxidized forms of A2E in culture media, cells or whole eyes was assessed by direct introduction of samples into the MS source. This allows a semi-quantitative estimate of the different oxidized forms in the samples. The MS scan analysis was performed by triple quadrupole mass spectrometer (API365, Applied Biosystems) operated in selected-ion monitoring positive-ion mode. The compounds with m/z values of 592, 608 and 624 were selected for subsequent MS scan in single-ion monitoring mode. The relative abundance of oxidized forms of A2E was obtained by calculating the ratio of AUC of oxidized forms of A2E (m/z 608, 624) and AUC of A2E (m/z 592).

### Pharmacokinetic studies of bixin and norbixin in mice

Two sets of pharmacokinetic studies were performed using C57BL/6J mice using bixin and/or norbixin. In the first set, bixin or norbixin were administered either *per os* (50 mg/kg, dissolved in a 1:9 mixture of DMSO and Isio4 oil) or intra-peritoneally (5 mg/kg, dissolved in 10%DMSO/20%Tetraglycol/70%H_2_O). In the second set, only norbixin was used and was dissolved in 10%DMSO/20%Tetraglycol/70%H_2_O for both p.o. (50 mg/kg) and i.p. (5 mg/kg) administrations. Blood samples were collected after 0.25, 0.5, 1, 3, 6, 8 and 24 hours, centrifuged, and plasma samples were kept at -20°C until analysis. Eyes were dissected after 1, 3, 6 and 24 hours and immediately frozen. Bixin, norbixin and their metabolites were analyzed by HPLC-MS/MS.

### Analysis of mice plasma samples and eyes for bixin and norbixin content

HPLC analysis was performed on an Agilent 1200 with DAD. Bixin and norbixin were eluted from a reverse-phase C18 column (2.1x50 mm; 5 μm particles; Purospher Star, Merck, Molsheim, France) with the following gradient of acetonitrile in water (containing 0.1% formic acid): 0–90% (1.5 min), 90% (1 min), (flow-rate: 0.5 mL/min) and they were monitored at 460 nm. For quantification of bixin and norbixin, a calibration curve was performed under the same conditions of the matrix of samples, with various amounts of bixin and norbixin (10–50000 ng/mL).

Plasma samples (30 μL) from different animals, and methanol (100 μL) were distributed in a 96-well microtitre plate, mixed for 10 min and precipitated. The microtitre plate was frozen at -20°C for 30 min and then centrifuged. The hydro-alcoholic phase was removed from each well and transferred into another microtitre plate for LC-MS/MS analysis. Under these conditions, with 20 μL injections, the limit of quantification (LOQ) was 50 ng/mL (= *ca*. 2.5 pmol). Eye samples were treated with the same protocol as for A2E measurements (see above).

Norbixin isomers were analyzed by LC-MS/MS on an Agilent 1200 with DAD and in-line triple quadrupole mass spectrometer (6420, Agilent, Courtabœuf, France) operated in MRM positive-ion mode. HPLC used a reverse-phase C18-column (2.1x50 mm; Fortis-18) eluted with the following gradient of acetonitrile in water (containing 0.1% formic acid): 60–95% (2.5 min), 95% (2 min), (flow-rate: 0.3 mL/min). Norbixin and its isomers or metabolites (= glucuronides) were monitored at 460 nm and MRM mode with precursor ion/product ion ratio (381.1/144.9).

### Statistical analyses

For statistical analyses, Student’s t-Test or one-way ANOVA followed by Dunnett’s or Tukey’s tests were performed using Prism 5 (GraphPad Software, La Jolla, CA, USA) depending of the sample size.

## Results

### Bixin and norbixin protect RPE cells against A2E-induced phototoxicity *in vitro*

In order to mimic lipofuscin-mediated photooxidation *in vivo* we developed an acute model of phototoxicity using porcine primary RPE cells loaded with 30 μM A2E and illuminated with blue light (Fig 2A). Under these conditions we obtained 60 to 70% cell death (Fig 2B and 2C) compared to control cells. Bixin or norbixin, used at 20 μM, were able to highly protect RPE cells compared to cells treated with A2E (cont + A2E), as seen in Fig 2B and 2C. The photoprotection reached 89.5% and 83%, respectively, for bixin and norbixin, compared to 100% for RPE cells illuminated, but not treated with A2E. Only 36.5% RPE cells treated with A2E and illuminated survived the phototoxicity.

### Bixin and norbixin are more efficient than lutein, zeaxanthin and crocetin

**Fig 3:**
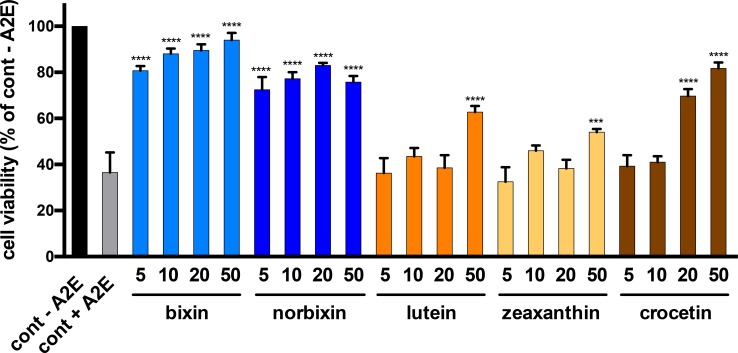
Dose-dependent effect of bixin, norbixin, lutein, zeaxanthin and crocetin on RPE cell survival after A2E-induced blue-light phototoxicity. Molecules were tested as described in Fig 2A. The concentrations of the substances are in μM. The positive control (cont–A2E) represents cells treated with DMSO alone. The negative control (cont + A2E) represents cells treated with A2E, but not with substances. Bars represent mean +/- s.e.m. of five independent experiments with *n* = 4. ***p<0.001; ****p<0.0001 compared to cont + A2E (One-way ANOVA, Dunnett’s post test).

The protective effect of bixin and norbixin was compared to those of three other carotenoids: lutein, zeaxanthin and crocetin. The five molecules were tested on the cellular RPE phototoxicity model at four concentrations (5, 10, 20, and 50 μM). As shown in Fig 3, bixin and norbixin were highly protective even at 5 μM, inducing 80.75 and 72.5% RPE survival, respectively, whereas lutein, zeaxanthin and crocetin showed no protection at this concentration. Lutein and zeaxanthin induced a significant protective effect at 50 μM with 62.75% and 54% cell survival compared to 36.5% for the non-treated control (cont + A2E). Crocetin was more efficient with a significant protective effect at 20 μM and 50 μM (69.75 and 81.75% cell survival, respectively).

### Norbixin is more bioavailable than bixin

**Fig 4:**
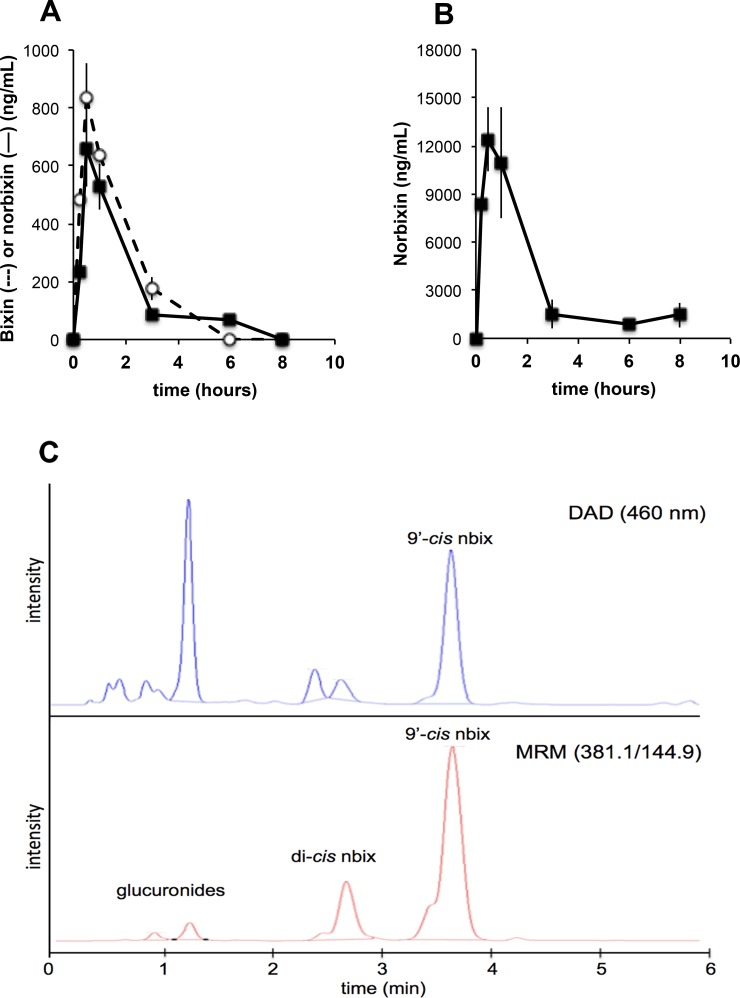
**Comparative pharmacokinetics of bixin (A) and norbixin (B) given orally.** (A) bixin or (B) norbixin were given at 50 mg/kg. Plasma analyses—note that bixin is efficiently converted into norbixin (values are the mean of three different animals). **(**C) HPLC-MS/MS analysis of a mouse plasma sample following oral administration of 9’-*cis*-norbixin. Both norbixin isomers and glucuronide conjugates are observed. DAD: diode-array detector; MRM: multiple reaction monitoring.

Pharmacokinetic studies performed with bixin and norbixin in mice showed that (1) bixin was much less bioavailable than norbixin and (2) bixin was efficiently converted into norbixin owing to esterase activities in both intestine and plasma (Fig 4A and 4B). The overall oral bioavailability of bixin (i.e. bixin + norbixin) was at best 15–20 times lower than that of norbixin (a similar result was obtained in rats [59]), and this result prompted us to focus on norbixin for all subsequent *in vivo* experiments.

A second set of pharmacokinetic studies with norbixin was performed using chromatographic analyses with greater resolution, which showed that the administered 9’-*cis*-norbixin underwent a significant isomerization (Fig 4C) to probably the all-*trans*- and one di-*cis-*isomer(s), on the basis of their HPLC behaviour [60]; their full identification using nuclear magnetic resonance is in progress. The proportion of these isomers increased with time and they represented *ca*. 50% of the total norbixin 6 hours after administration. In addition, two more polar compounds were also detected in the Multiple Reaction Monitoring (MRM) mode. They were shown to have a molecular mass of 566 a.m.u., and they disappeared upon glucuronidase treatment. These two peaks are thus expected to be monoglucuronides of norbixin.

In the second experiment, the oral bioavailability of norbixin was close to 96% (data not shown). Plasma norbixin levels peaked at *ca*. 20 μM after 15 min and remained above 5 μM for at least 6 hours after administration. Eye uptake was rather low (1–2 ng/eye), but some norbixin (1 ng/eye) was still found unchanged 24 hours after a single administration, together with glucuronides.

### Development of a blue-light damage model using the *Abca4^−/−^ Rdh8^−/−^* mouse model

**Fig 5:**
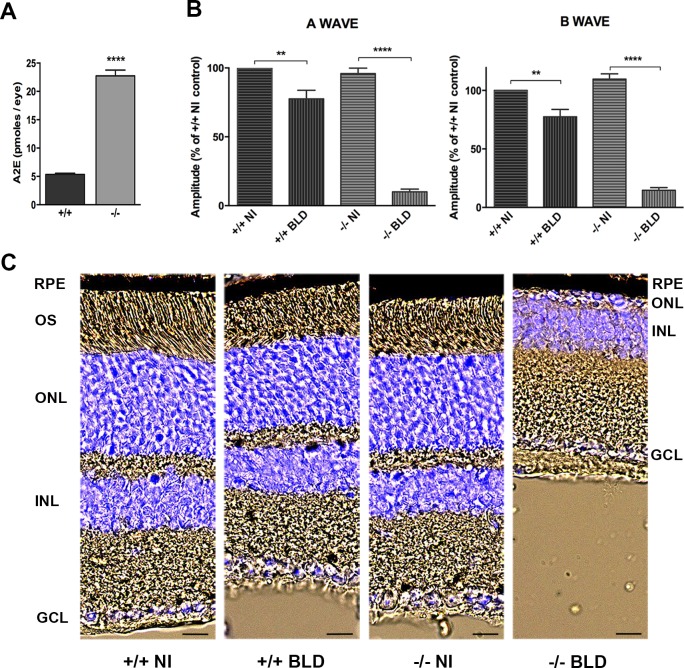
Effect of BLD on *Abca4^-/-^ Rdh8^-/-^* and *Abca4^+/+^ Rdh8^+/+^* mice carrying the Rpe65-Leu450 and the *rd8* mutations. (A) A2E quantification in eyes from young (7–8 weeks) *Abca4^+/+^ Rdh8^+/+^* (+/+) and *Abca4*^*-/-*^ *Rdh8*^*-/-*^ (-/-) mice. Bars represent mean ± s.e.m. of 3 eyes. ****p<0.0001 (Student’s t Test). (B) BLD was induced in +/+ and -/- during 1 hour and ERGs were recorded 7 days later. Non-illuminated +/+ mice were used as controls. A- and b-wave amplitudes are presented for the four groups and expressed as percentage of the non-illuminated *+/+* control. Bars represent mean ± s.e.m. of two separate experiments with *n* = 3–6. **p<0.01 ****p<0.0001 (One-way ANOVA, Tukey’s post-test). (C) Representative cryosection pictures showing Hoechst 33342 staining of the retinal cell nuclei one week after BLD. NI: non-illuminated. Scale bars = 20 μm. OS: outer segment; ONL: outer nuclear layer; INL: inner nuclear layer; GCL: ganglion cell layer.

To test the efficacy of norbixin *in vivo*, we developed a model of BLD using 7-week-old *Abca4^−/−^ Rdh8^−/−^* mice, an animal model of dry AMD [52] accumulating A2E in RPE cells. In order to see whether A2E is involved in the retinal degeneration induced by BLD we used *Abca4*^+/+^ *Rdh8*^+/+^ mice (see materials and methods), in which A2E does not accumulate in RPE cells as fast as in *Abca4^−/−^ Rdh8^−/−^* mice (Fig 5A). At the age of 7 weeks, both *Abca4^−/−^ Rdh8^−/−^* and *Abca4*^+/+^ *Rdh8*^+/+^ mice displayed a normal ERG (Fig 5B) and did not show any retinal damage (Fig 5C). In contrast, one-hour BLD induced a strong decrease of ERG in the knockout mice compared to the *Abca4*^+/+^ *Rdh8*^+/+^ mice. A- and b-wave amplitudes were decreased by 90% and 87%, respectively, in the knockout mice, whereas they only decreased by 22% and 23% in the *Abca4*^+/+^ *Rdh8*^+/+^ mice. ERG decrease was accompanied by a massive loss of photoreceptors in the *Abca4^−/−^ Rdh8^−/−^* retina, whereas there was no apparent cell degeneration in the *Abca4*^+/+^ *Rdh8*^+/+^ retina. These results show that the retinal degeneration observed in *Abca4^−/−^ Rdh8^−/−^* mice after BLD is probably linked to the accumulation of A2E.

### Norbixin protects the retina of *Abca4*^*-/-*^ *Rdh8*^*-/-*^ mice against blue-light-induced phototoxicity

**Fig 6:**
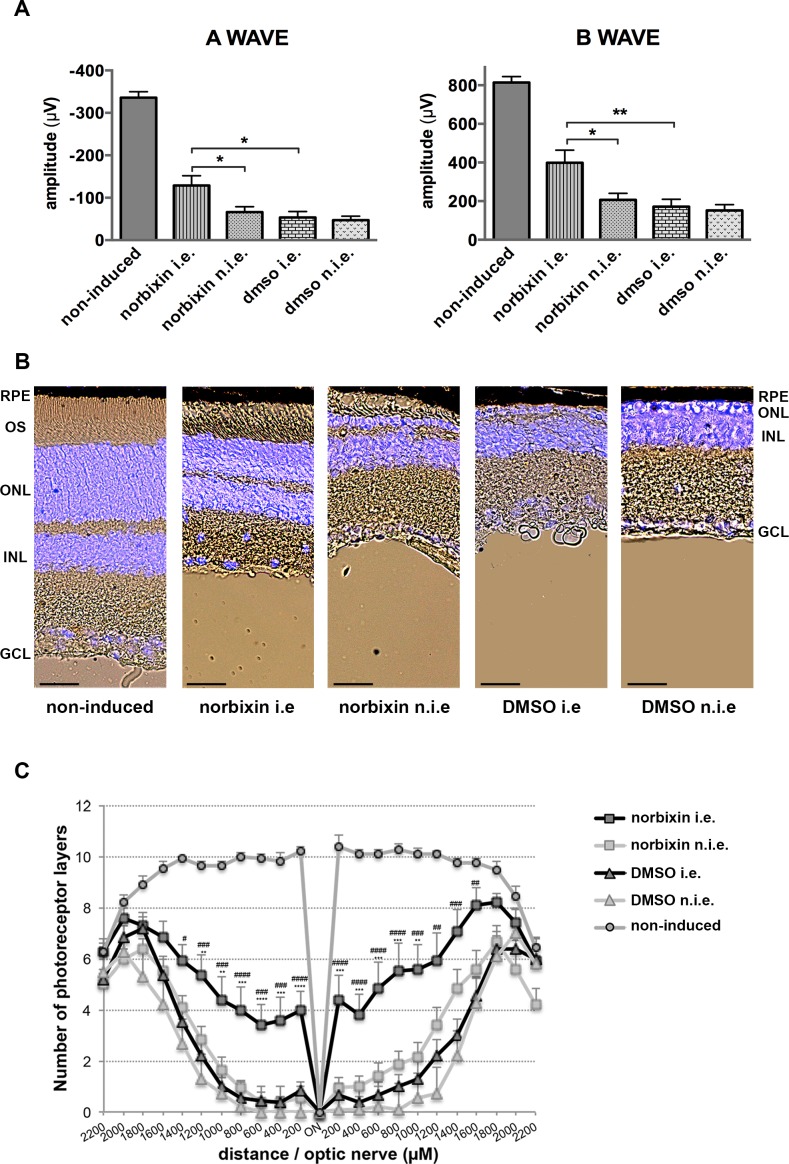
Effect of norbixin on ERG and retinal phototoxicity after BLD in the *Abca4*^*-/-*^ *Rdh8*^*-/-*^ mouse. (A) ERG from *Abca4*^*-/-*^ *Rdh8*^*-/-*^ mice injected in one eye with either norbixin or vehicle and light-exposed were recorded after 7 days. A- and b-wave amplitudes are presented for the five groups studied. non-induced: non-injected and non-illuminated; i.e.: injected eye; n.i.e.: non-injected eye. (B) Representative cryosection pictures showing Hoechst 33342 staining of the retinal cell nuclei one week after BLD in norbixin- or DMSO-injected or non-injected eyes and compared to non-induced eyes. OS: outer segment; ONL: outer nuclear layer; INL: inner nuclear layer; GCL: ganglion cell layer. Scale bars = 25 μm. (C) Graph showing the number of photoreceptor layers measured along the retina each 200 μm from the optic nerve. *: norbixin i.e. compared to norbixin n.i.e.; ^#^: norbixin i.e. compared to DMSO i.e. Data from (A) and (C) represent the mean ± s.e.m. of four separate experiments with *n* = 3–4. (A): *p<0.05, **p<0.01 (One-way ANOVA, Tukey’s post test). (C) ^#^ *p*<0.05, ** or ^##^*p*<0.01, *** or ^###^*p*<0.001, **** or ^####^*p*<0.0001 (One-way ANOVA, Dunnett’s post test).

To evaluate the therapeutic effect of norbixin, we used the BLD model described above and *Abca4*^*-/-*^ *Rdh8*^*-/-*^ mice were injected in one eye 24 hours before BLD. In a first set of experiments 2 μL of 75 μM norbixin were injected in one eye (corresponding to a final intravitreal concentration of 20 μM). At this concentration we observed a slight, but not statistically significant, effect on ERG or photoreceptor survival (data not shown). In a second set of experiments 2 μL of 500 μM norbixin were injected in order to reach an intravitreal concentration of 130 μM. Mice were then kept in the dark until BLD. Single-flash ERGs were recorded under scotopic conditions one week later. Mice injected with dimethylsulfoxide (DMSO) alone served as negative controls. As seen in Fig 6A, a- and b-wave amplitudes were maintained significantly better in norbixin-injected eyes than in DMSO-injected eyes or in non-injected eyes. Retinal cryosection and Hoechst 33342 staining were used to evaluate retinal degeneration and the ONL thicknesses were measured all along the retina. In non-injected eyes of mice, ONL thickness was markedly decreased with only 0 to 1 row of photoreceptors left in the central light-damaged retina and outer segments were absent (Fig 6B and 6C). In the eyes injected with norbixin, we observed a partial, but clear, protection of photoreceptor cells and outer segments compared to the contralateral eyes of the same mice or to the DMSO-injected eyes (Fig 6B). In the central retina, 4 to 6 rows of photoreceptors were still present one week after BLD compared to 1–2 rows for DMSO or norbixin non-injected eyes (Fig 6C).

### Norbixin protects the retina of albino rats against blue-light-induced phototoxicity

**Fig 7:**
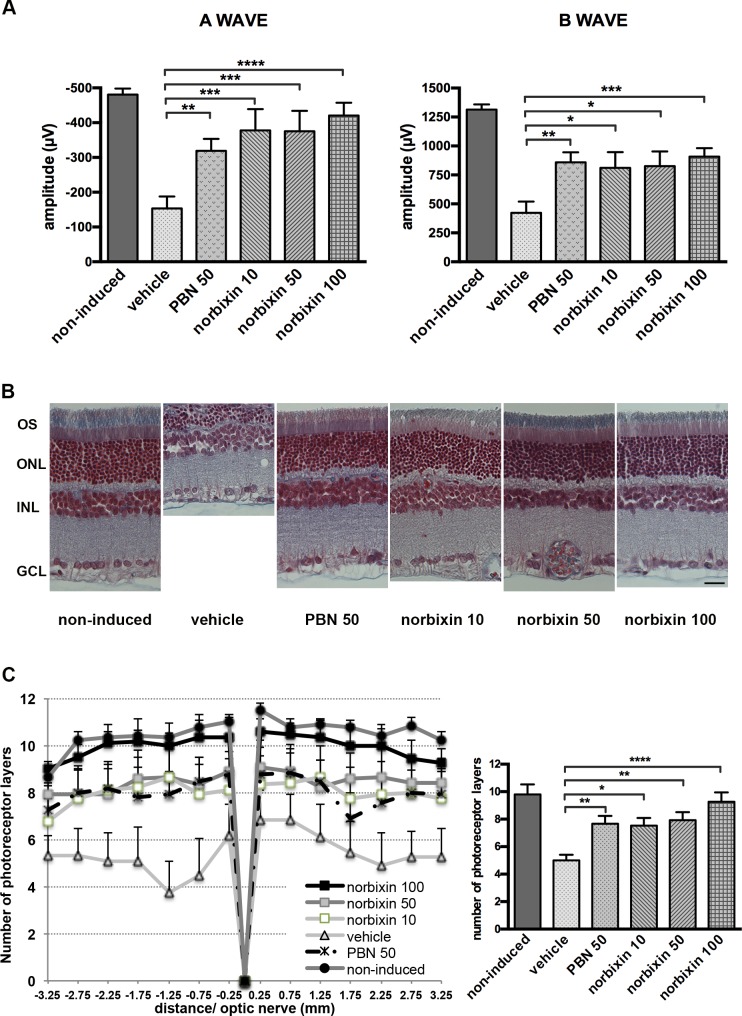
Effect of norbixin on ERG and retinal phototoxicity after BLD in the rat. ERG from rat non-induced or light-exposed which received vehicle, norbixin or PBN. Recordings were on recovery day 7. (A) A- and b-wave amplitudes are presented for the six groups studied. (B) Representative pictures of retinal sections stained with Trichrome-Masson showing cell nuclei one week after BLD. Scale bars = 30 μm. (C) Morphometric analysis of retina. Photoreceptor cell nuclei were measured from the optic nerve to the superior and inferior side of the retina. Data from (A) and (C) represent the mean ± s.e.m. of two separate experiments with *n* = 3. *p<0.05, **p<0.01, ***p<0.001, ****p<0.0001 compared to vehicle-treated group (One-way ANOVA, Dunnett’s post test).

To determine whether norbixin could protect the retina via a systemic effect we used a model of rat BLD, and norbixin (10, 50 or 100 mg/kg body weight) was injected intraperitoneally prior to BLD and 2, 4 and 6 hours after the beginning of the exposure. PBN (50 mg/kg), a potent free-radical trapping agent known to protect the retina in this model [61,62] was used as a positive control. Six hours of blue-light exposure induced severe retinal damage in vehicle-dosed rats, as measured by ERG (Fig 7A). Seven days after exposure, the a- and b-wave amplitudes were -481 ± 17 μV and 1314 ± 44 μV in control rats, compared with -154 ± 34 μV and 424 ± 97 μV in light-exposed eyes from vehicle-treated rats. In the light-exposed rats, administration of norbixin (10, 50 and 100 mg/kg) protected against the reduction of a-wave amplitude by 69, 68 and 81%, respectively (p<0.001 and p<0.0001 *vs* vehicle), whereas 50 mg/kg of PBN induced a 50% protection. The decrease in b-wave amplitude was also significantly reduced by norbixin and PBN with a maximum of 57% protection in rats treated by 100 mg/kg norbixin. To confirm the protective effect of norbixin, morphological evaluation of the retina was performed. The number of photoreceptor cell nuclei was measured along the retina and values were plotted as a function of the distance from the optic nerve (Fig 7B). After light exposure, a loss of photoreceptors was observed in retinal sections of light-exposed eyes from vehicle-treated rats. Treatment with 10 and 50 mg/kg of norbixin partially protected photoreceptors similar to the reduction provided with PBN (Fig 7B and 7C). For the rats treated with 100 mg/kg of norbixin, the ONL was preserved and 95% photoreceptors remained (Fig 7C).

### Norbixin reduces A2E accumulation in RPE of *Abca4*^*-/-*^ *Rdh8*^*-/-*^ mouse *in vivo*

**Fig 8:**
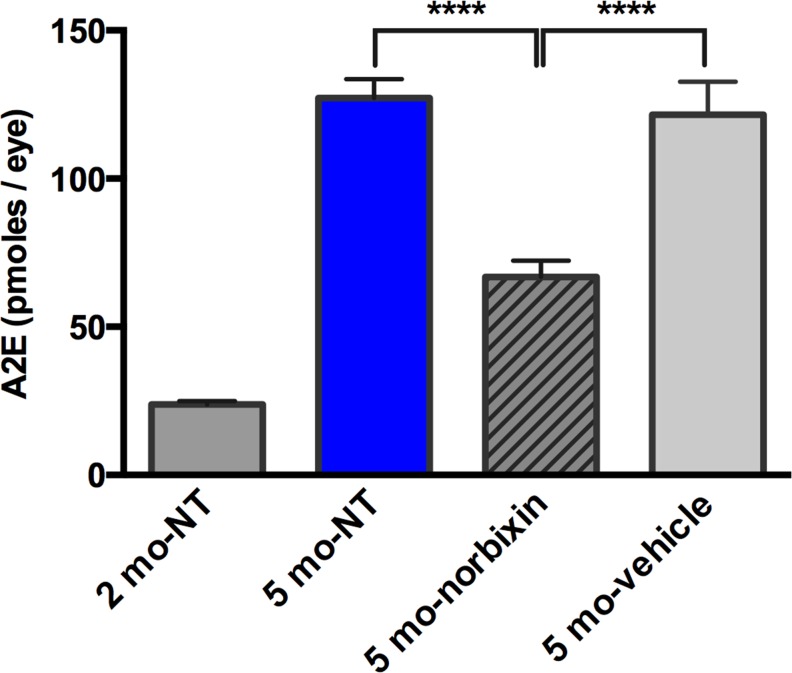
A2E quantification after norbixin water supplementation. Two-month-old *Abca4*^*-/-*^ *Rdh8*^*-/-*^ mice were treated for 3 months with drinking water supplemented with 0.5 mg/mL norbixin or vehicle. Eyes from five-month old and two-month old *Abca4*^*-/-*^ *Rdh8*^*-/-*^ mice were also analysed and served as controls. NT: non treated. Data represent the mean +/- s.e.m. of 2 separate experiments with *n* = 5. ****p<0.0001 compared to norbixin-treated mice (One-way ANOVA, Dunnett’s post test).

*Abca4^−/−^ Rdh8^−/−^* mice are characterized amongst other things by an elevation of A2E in RPE cells owing to an abnormal functioning of the visual cycle (Maeda et al., 2008). In order to see whether chronic treatment with norbixin could reduce A2E accumulation in these mice, we supplemented their water-beverage during 3 months before removing eyes for A2E quantification (Fig 8). A2E content in RPE of five-month old mice increased 5-fold compared to two-month old mice (127 pmol/eye *vs* 24 pmol/eye) showing a rapid increase over 3 months. In norbixin-treated mice A2E quantity represented only 55% of that found in vehicle-treated mice (67 pmol/eye *vs* 122 pmol/eye).

### Norbixin reduces A2E accumulation by RPE cells *in vitro*

**Fig 9:**
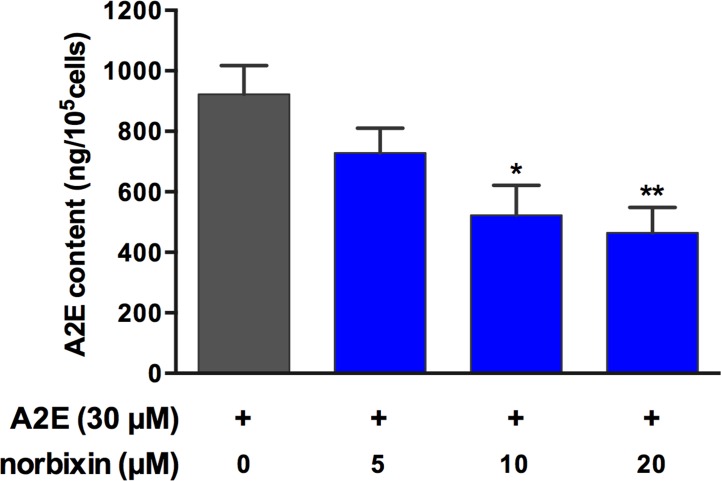
Effect of different concentrations of norbixin on the accumulation of A2E by porcine RPE cells *in vitro*. RPE cells were treated with norbixin (or DMSO) and with A2E but not illuminated and cells were extracted with MeOH. A2E concentrations are expressed as absolute amounts per 10^5^ cells. Bars represent the mean ± s.e.m. of three separate experiments with *n* = 3. **p<0.01, ***p<0.001 compared to cells non treated with norbixin (One-way ANOVA, Dunnett’s post test).

Porcine RPE cells were treated with A2E and various concentrations (5, 10, and 20 μM) of norbixin, according with the protocol described in Fig 2A. The amounts of A2E accumulated within RPE cells, as determined by HPLC-MS/MS, were much reduced in the presence of norbixin (Fig 9). At the same time, we also analysed the presence of toxic oxidized forms of A2E by MS, both in the media and within cells. A significant oxidation of A2E was observed in media and it was even higher in cells (Table 1). There was almost no difference of the proportions of oxidized forms between control and norbixin-treated samples relative to non-oxidized A2E (Table 1)–but given their lower concentrations of A2E, it can be concluded that cells treated with norbixin contain lower amounts of mono- and di-oxidized forms of A2E.

**Table 1:** Relative abundance of mono- and di-oxidized forms of A2E in cells and media: effect of norbixin.

Sample	Oxidized forms in cells (%)		Oxidized forms in media (%)	
	608/592	624/592	608/592	624/592
DMSO control	14.3±1.7	7.4±0.8	6.8±0.4	1.4
Norbixin 5 μM	17.4±2.3	8.7±1.9	7.3±0.7	1.5
Norbixin 10 μM	19.5±2.3	10.5±2.2	8.4±0.7	1.4
Norbixin 20 μM	15.6±2.3	7.5±0.8	8.8±0.7	1.5

Relative abundance of mono- and di-oxidized forms (respectively ions at m/z 608 and 624) of A2E (ion at m/z 592) in cells and media in the presence or absence of norbixin in culture media (mean ± s.e.m. of three experiments, each in triplicate).

## Discussion

The present study demonstrated the high photoprotective effect of norbixin and bixin at low concentration on primary cultures of porcine RPE cells exposed to A2E and blue light. Norbixin administrated by intravitreal (120 μM) or intraperitoneal (10, 50 and 100 mg/kg) injections protected the retina from an acute blue-light damage in two different models *in vivo*, and, upon chronic oral administration, it reduced A2E accumulation in a mouse model of dry AMD.

### Bixin and norbixin protect RPE cells *in vitro*

RPE cell cultures are widely used for AMD studies for testing drug candidates or analysing the molecular mechanisms of AMD [63]. Either primary cell cultures or RPE cell lines (especially human ARPE19 cells) are used. We may wonder whether established cell lines still behave as *in situ* RPE cells, as they do not express the same cellular receptors [64,65].

In the present study, RPE cells cultivated in the presence of A2E, and then exposed to blue light provided a reproducible model of RPE phototoxicity. Schütt et al. showed a significant loss of cell viability by 72 hours after light exposure of A2E-treated RPE cells [66]. Sparrow et al. demonstrated that ARPE19 cells preloaded with A2E underwent cell death when exposed to blue light [67]. The number of nonviable cells varied with duration of light exposure, concentration of A2E, and time after light exposure. In our case, the blue light treatment was calibrated to allow a rather high (40%) percentage of A2E-treated cells survival, which might explain that we detect effects with low concentrations of protective substances.

Most in vitro studies consider RPE cells protection against an oxidative stress (H_2_O_2_, tBuOOH) rather than A2E exposure, and the authors measure either cell survival or the production of ROS or of malondialdehyde (MDA). We may however wonder whether these different models are equivalent. Indeed, vitamin C was efficient in protecting against tBuOOH, but not against H_2_O_2_ [50]. Similarly, anthocyanidins did not protect against H_2_O_2_ or tBuOOH [68], whereas they were efficient against A2E [69].

### Bixin and norbixin are more efficient than other carotenoids

Bixin and norbixin display a significant protection at concentrations as low as 5 μM, which is not the case for lutein and zeaxanthin. To confirm that these molecules are intrinsically more active and the difference of efficient dose was not related to a difference of carotenoid uptake, we measured the amounts of carotenoids remaining in solution or attached to microplate wells after 24 hours (unpublished data). In the absence of cells, 85% of lutein and 64% of zeaxanthin bound to plastic and could be removed with alcohol, versus 8% for bixin and norbixin. In the presence of RPE cells, binding to plastic was reduced (58% for lutein and 51% for zeaxanthin). This certainly contributes to lowering the efficiency of xanthophylls, although this does not suffice to explain the difference with bixin/norbixin.

### Norbixin pharmacokinetics

Orally administered norbixin is rapidly and efficiently absorbed, and this confirms previous data with rats [59]. The ingested compound (the 9’-*cis* isomer) undergoes significant isomerization and glucuronidation (Fig 4C). Glucuronide conjugation has also been described for crocetin [70]. When crocetin was given orally to rats (100 mg/kg), a plasma concentration of 109.6 μM was observed after 1 hour [71], consistent with our findings for norbixin in mice. It is expected that similar reactions will take place in humans, and indeed norbixin was detected in human plasma up to 16 h after ingestion of a single dose of bixin, with a T_max_ of 2–4 hours [72], comparable to that of crocetin (4 hours) [73].

### Norbixin protects the retina *in vivo*

*Abca4*^*-/-*^ *Rdh8*^*-/-*^ mice represent a model of dry AMD as they accumulate high amounts of A2E in their eyes and are therefore more susceptible to BLD [52]. All-*trans*-retinal accumulation activates the proapoptotic factor Bax and evokes retinal cell death [74]. Light-induced (10,000 lux for 1 hour) retinal degeneration in this model demonstrated the efficacy of drugs such as retinylamine or emixustat [52,75,76]. By contrast, this treatment induced no retinal degeneration and only a slight ERG loss in *Abca4^+/+^ Rdh8^+/+^* Rpe65-Leu450 and *rd8* background mice. These results provide evidence that A2E accumulation and retinal degeneration are tightly connected and, consequently, that norbixin protection could occur through counteracting its deleterious effects.

Intravitreal injections of norbixin afforded good protection. However, this route of administration is not suitable in clinical practice, as the expected half-life of norbixin is too short to allow monthly treatments. Indeed, norbixin was hardly detectable in mouse eyes 24 hours after intravitreal injections (unpublished data).

In the rat blue-light model. norbixin proved at least as efficient as PBN (Fig 7). Similarly, an efficient protection of the skin against UV was observed 48 hours after a single intraperitoneal injection of bixin (200 mg/kg) in rats [77].

Bixin (this probably also applies to norbixin) is also able to protect retinal ganglion cells (RGC) against an endoplasmic reticulum stress induced by intravitreal injections of tunicamycin, as well as tunicamycin-treated RGC cells *in vitro* [78]. Similarly, crocetin prevented RCG death from endoplasmic reticulum stress [71].

### Chronic oral supplementation with norbixin

Chronic oral supplementation with norbixin resulted in a reduced accumulation of A2E in mice eyes. There is a general consensus that reducing A2E formation and/or accumulation is a worthwhile target for pharmacological interventions against AMD. Although severe light restriction reduces A2E formation in animal models [79], this approach is probably not practical in humans.

Blocking the visual cycle to limit the density of visual pigment and in turn production of trans-retinal and A2E was also investigated. Several inhibitors of the visual cycle have been tested, but they cause night-blindness, which may be uncomfortable for the patients, and retinoid-based compounds can cause significant systemic effects and teratogenicity [80,81,82]. Other therapeutic strategies targeting RPE bisretinoids such as scavengers of all-*trans*-retinal [83], or molecules able to degrade [34] or remove A2E from RPE cells [84,32] have been proposed, but additional research is required to demonstrate their safety and efficacy in humans. Lutein and zeaxanthin, which are naturally present in the human macula, decreased A2E levels in RPE of Japanese quails after 16 weeks of supplementation [36]. Ramkumar et al. showed that *Ccl2*^*-/-*^ *Cx3cr1*^*-/*-^ mice fed for 3 months with AREDS2 formula containing lutein and zeaxanthin had lower concentrations of A2E compared to the non-supplemented group [85]. How lutein and zeaxanthin, as well as norbixin, are able to reduce the accumulation of A2E in RPE cells remains to be elucidated.

### What might be the mechanisms of action of norbixin?

A2E accumulation has deleterious effects that may proceed directly, owing to its cationic detergent properties, or indirectly after through its photo-oxidation by-products. A2E impairs lysosome and proteasome activity [35] and initiates inflammation processes [8]. A2E involvement in retinal degeneration is further assessed by its deleterious effects after intravenous injections in rabbits [86].

Norbixin reduced A2E accumulation by RPE cells *in vitro* (Fig 9). Whether it reduces A2E uptake or stimulates some process of transport outside the cells is presently investigated using monolayers of RPE cells cultivated in transwell cell culture inserts.

One possible protection mechanism involves the activation of Nrf2 and the subsequent production of phase II detoxification enzymes (e.g. superoxide dismutase SOD). Such enzymes protect the retina against oxidative stress, and animals where SOD gene is inactivated develop AMD-like signs of retinal degeneration [87,88]. There is indeed good evidence that Nrf2 is involved in the protection of RPE against degeneration [89]. Nfr2 activation was described for lutein [90,91], zeaxanthin [92], astaxanthin [93] and bixin [77], but these effects were not always observed with RPE cells, so they may require confirmation using appropriate target cells.

Primary target(s) should possibly be considered among PPAR receptors, which accept a great variety of ligands and could play a key role in the prevention of AMD [94]. In other cell systems, some effects of bixin and/or norbixin involve their binding to PPAR receptors [95,96,97]. This hypothesis deserves more detailed studies and is presently under investigation.

### Conclusion

Norbixin appears promising for developing an oral macular degeneration treatment. This molecule is already used in human food as a natural dye and its lack of toxicity established in many assays (e.g. [98]). A drug candidate (BIO201) based on highly purified 9’-*cis*-norbixin as active principle ingredient is under development, and its potential will be assessed in a forthcoming multicentre clinical trial.

## Abbreviations

A2E: *N*-retinylidene-*N*-retinylethanolamine
AMD: age-related macular degeneration
AUC: area under curve
BLD: blue-light-induced retinal damage
DMSO: dimethylsulfoxide
ERG: electroretinogram
HPLC: high-performance liquid chromatography
HPLC-MS/MS: HPLC coupled with tandem mass spectrometry
MRM: Multiple Reaction Monitoring
ONL: outer nuclear layer
OS: outer segments
INL: inner nuclear layer
GCL: ganglion cell layer
PBN: Phenyl-N*-tert*-Butylnitrone
PBS: phosphate buffer saline
PPAR: Peroxisome proliferator-activated receptor
RPE: retinal pigment epithelium
